# Is interval chemotherapy safe and does it improve the outcome of patients with colorectal liver metastases undergoing multimodal two-stage hepatectomy? – A systematic literature review

**DOI:** 10.1186/s12885-024-13008-9

**Published:** 2024-10-10

**Authors:** Nathanael Raschzok, Simon Moosburner, Moritz Blank, Felix Krenzien, Georg Lurje, Wenzel Schöning, Igor M. Sauer, Johann Pratschke, Dominik P. Modest, Annika Kurreck

**Affiliations:** 1 Department of Surgery, Campus Charité Mitte | Campus Virchow-Klinikum, Charité – Universitätsmedizin Berlin, Corporate member of Freie Universität Berlin and Humboldt-Universität zu Berlin, Berlin, Germany; 2 Department of Hematology, Oncology, and Cancer Immunology (CCM/CVK), Charité – Universitätsmedizin Berlin, Corporate member of Freie Universität Berlin and Humboldt-Universität zu Berlin, Berlin, Germany; 3 Berlin Institute of Health at Charité - Universitätsmedizin Berlin, BIH Academy, Clinician Scientist Program, Berlin, Germany

**Keywords:** Two-stage hepatectomy, Staged hepatectomy, Interval chemotherapy, Systematic therapy, Colorectal liver metastases, Systematic literature review

## Abstract

**Background:**

Multimodal two-stage hepatectomy (mTSH) is used in patients with bilobar colorectal liver metastases (CRLM) that cannot be treated with one surgical procedure due to insufficient future liver remnant. Interval chemotherapy has been proposed to improve disease control in CRLM patients undergoing mTSH. We here present a narrative review of clinical studies on mTSH including the use of interval chemotherapy in patients with CRLM.

**Methods:**

A systematic literature search of the PubMed databases as well as the ClinicalTrials.gov registry was performed.

**Results:**

The use of interval chemotherapy during mTSH was reported in 23 studies and applied in 595 out of 1,461 patients with CRLM. Two studies report on the actual effects of this treatment, one study describes a trend towards improved disease progression rate. No serious adverse events caused by interval chemotherapy were observed. There is currently no randomized clinical trial investigating the efficacy and safety of interval chemotherapy during mTSH.

**Conclusion:**

The currently available data indicate that interval chemotherapy does neither impair liver hypertrophy during mTSH nor cause procedure-associated complications in patients with CRLM. Results from randomized clinical trials on the potential positive effect on disease control are not yet available.

## Background

**Fig. 1:**
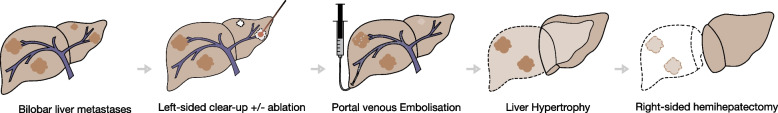
Graphical abstract. Multimodal two-stage hepatectomy involves the initial stage, which includes surgical and optionally interventional clearance of the future liver remnant through ablation, typically focusing on the left lobe. This is followed by hypertrophy induction in the future liver remnant by using portal vein embolization or portal vein ligation. The second step, which is generally performed after a waiting period of 4-8 weeks to ensure a sufficient future liver remnant, includes the final surgical resection of all remaining tumor-bearing areas of the liver

Colorectal cancer (CRC) is the third most diagnosed cancer worldwide [1]. Approximately 40-50% of all CRC patients either have metastases at time of their initial diagnosis or develop metastasis during the course of the disease [2]. Among patients with metastatic colorectal cancer (mCRC), about 15-30% are eligible for local treatment approaches that have been shown to enhance survival [3]. There is additional evidence suggesting that these patients gain an even greater benefit from multimodal treatment strategies including systemic antitumor therapy [2, 4]. The liver is the most prevalent site for metastatis in CRC. The current standard of care involves the removal of all colorectal liver metastases (CRLM) through surgical resection, ablation, or radiation [2]. In cases where patients with bilobar CRLM cannot be treated with a single surgical procedure due to an insufficient future liver remnant (FLR), a multimodal two-stage hepatectomy (mTSH) may be considered. The first step includes surgical and optionally interventional clearance of the future liver remnant by using ablation, usually the left lobe, followed by hypertrophy induction in the FLR by portal vein embolization (PVE) or portal vein ligation (PVL). After a waiting period of 4-8 weeks to obtain a sufficient FLR the second step of the final surgical resection of all remaining tumor-bearing areas of the liver takes place (Fig. 1). However, it is noteworthy that 20-40% of patients undergoing mTSH do not complete the second-stage resection, primarily due to intrahepatic tumor progression [5].

Systemic antitumor therapy is used with the intention to prevent cancer progression. Discontinuing such treatment during active disease can lead to tumor progression [6]. Fisher et al. have shown that chemotherapy following PVE to induce liver hypertrophy prior to major hepatectomy can prevent from tumor growth in patients with CRLM without adverse effects on liver hypertrophy [7]. Accordingly, interval chemotherapy between first- and second-stage resection has been used to improve disease control in patients with biolobar CRLM undergoing mTSH by enabling a completion of the curative intended treatment algorithm [8]. However, neither results from randomized clinical trials nor data from meta-analyses are available in current literature to support this strategy.

The objective of this narrative review is to present a brief overview of the existing literature on interval chemotherapy during hypertrophy phase of mTSH focusing on potential negative effects, e.g. on liver hypertrophy, as well as possible positive effects on disease control in patients with CRLM.

## Methods

### Search strategy

A systematic literature review was conducted in accordance with the Preferred Reporting Items for Systematic Reviews and Meta-Analyses for Protocols 2015 (PRISMA-P). On October 19, 2022, and last updated on February 9, 2023, an extensive search of the National Library of Medicine Database and the ISI Web of Science Database was carried out to identify publications related to interval chemotherapy during the hypertrophy phase of mTSH.

The following search queries were performed:

1. “two-stage hepatectomy AND colorectal”
2. “staged hepatectomy AND colorectal”
3. “2-stage hepatectomy AND colorectal”
4. “ALPPS AND colorectal”

In addition, the ClinicalTrials.gov registry of the U.S. National Library of Medicine was searched on August 30, 2022 for the following MeSH term:

- “two-stage hepatectomy”

### Inclusion and exclusion criteria

Articles included in this review were clinical studies describing interval chemotherapy during mTSH for treatment of CRLM. Excluded were editorials, letters, reviews, case reports, conference abstracts, and video articles. Studies were excluded if they reported on patient populations with mixed entities where the identification of CRLM patients was not clearly recognizable.

Inclusion criteria for clinical trials involved any kind of trials on interval chemotherapy as part of mTSH for patients with CRLM with the following recruitment statuses: “recruiting”, “active, not recruiting”, “not yet recruiting” or “enrolling by invitation”.

### Data extraction

To increase transparency of the findings of this systematic review the whole viewing process has been conducted by two independent individuals (MB and SM). In instances of disagreement, the corresponding author NR was consulted, and consensus was reached through discussion. During the first stage of data extraction, the titles and abstracts of all retrieved articles were reviewed, and unsuitable studies were excluded. During the second stage, full text articles of remaining studies were screened carefully and assessed for inclusion criteria. For identification of clinical trials, the study description was carefully assessed. The extracted data were reviewed and analyzed by all authors.

### Statistical analyses

Statistical analyses were conduct using Microsoft Excel for Mac, Version 16.71. Unless otherwise specified, all presented data are expressed as mean ± standard deviation.

## Results

### General information

**Fig. 2:**
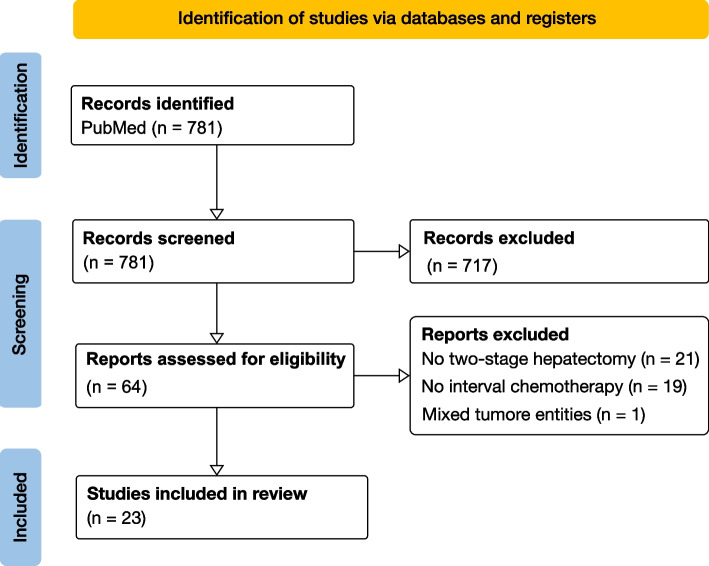
PRISMA statement

Systematic literature search of the National Library of Medicine database identified 781 unique records (Fig. 2). By using title and abstracts, 717 papers were excluded, resulting in 64 articles undergoing a full-text analysis. Among these, 40 publications did not meet our inclusion criteria, and one article reporting on findings from a mixed patient population in which CRLM patients could not be clearly identified.

Concerning currently registered studies, a search on ClinicalTrials.gov revealed a total of twelve trials. However, no active trial was listed addressing interval chemotherapy during mTSH.

### Two-stage hepatectomy with interval chemotherapy

Our literature search identified 23 studies published between 2012 and 2021 reporting on interval chemotherapy during the hypertrophy phase of mTSH (Table 1 [8–30]). PVE was used in all studies, PVL was additionally used in 10 out of these studies. Time between first- and second-stage resection was 2.7 ± 1.3 months. Ablation was performed in 57% of all studies (177 out of 1,461 patients). Tumor progression during the hypertrophy phase was reported in 15% of all patients, the mean dropout rate from second-stage resection was 18%. The overall mean morbidity rate was 42% (any complication), the mean mortality rate was 6.7%. A median overall survival (OS) of 39.2 months was reported with a median disease-free survival of 12.5 months resulting in a 1-/3-/5-year survival of 82%/53%/32% in patients completing the second stage of mTSH.

**Table 1:** Study Results

	**Chavez ** [8]	**Ibáñez** [9]	**Bednarsch ** [10]	**Robles-Campos ** [11]	**Quénet** [12]	**Okumaru** [13]	**Mor ** [14]	**Lillemoe** [15]	**Jouffret ** [16]	**Torzilli ** [17]	**Mizuno ** [18]	**Kikuchi ** [19]	**Griseri** [20]	**Cassinotto ** [21]	Passot [22]	Adam [23]	Fuks [24]	Giuliante [25]	Cardona [26]	Tanaka [27]	Dupré [28]	Stella [29]	Muratore [30]
**Year**	2021	2021	2020	2019	2019	2019	2019	2019	2019	2018	2018	2017	2017	2017	2016	2016	2015	2014	2014	2013	2013	2012	2012
**Patients intended for 2nd stage resection**^**#**^	195	15	37	41	56	86	29	137	68	74	126	20	43	40	109	41	34	130	40	24	13	56	47
**PVE/PVL**	PVE	PVE/PVL	PVE/PVL	PVE/PVL	PVE	PVE	PVE	PVE	PVE	PVE	PVE	PVE/PVL	PVE/PVL	PVE/PVL	PVE	PVE/PVL	PVE	PVE/PVL	PVE/PVL	PVE	PVE	PVE	PVE/PVL
**Ablation during/after 1**^**st**^** stage resection**^**#**^	0	n.a.	0	n.a.	25	n.a.	21	n.a.	39	9	n.a.	n.a.	9	n.a.	2	6	3	5	12	n.a.	7	37	0
**Time between 1**^**st**^** and 2**^**nd**^** stage resection**	4	2.1	1.9	1.2	6	n.a.	n.a.	n.a.	n.a.	n.a.	n.a.	1.6	2.07	2.1	n.a.	3.4	3.1	n.a.	n.a.	n.a.	1.7	2	3.8
**Tumor progression prior 2**^**nd**^** stage resection**^**#**^	n.a.	n.a.	1	2	16	n.a.	5	n.a.	13	30	28	n.a.	14	12	12	12	8	24	4	n.a.	2	5	25
**Dropout from 2**^**nd**^** stage resection**^**#**^	n.a.	4	3	2	21	n.a.	5	26	6	30	34	0	14	19	20	15	8	28	5	3	2	7	11
**Interval chemotherapy**^**#**^	118	n.a.	9	n.a.	50	34	9	n.a.	15	55	n.a.	2	36	n.a.	58	35	15	39	30	11	13	41	25
Chemotherapy Cycles (median)	n.a.	n.a.	n.a.	n.a.	6	n.a.	n.a.	n.a.	n.a.	n.a.	n.a.	n.a.	4.5	n.a.	n.a.	n.a.	n.a.	4	n.a.	4.5	2	2	4.4
Effect on Hypertrophy*	n.a.	n.a.	n.a.	n.a.	n.a.	n.a.	n.a.	n.a.	n.a.	n.a.	n.a.	n.a.	n.a.	n.a.	n.a.	n.a.	n.a.	n.a.	n.a.	n.a.	n.a.	n.a.	n.a.
Effect on Metastasis Growth*	n.a.	n.a.	n.a.	n.a.	n.a.	n.a.	n.a.	n.a.	n.a.	n.a.	n.a.	n.a.	n.a.	n.a.	n.a.	n.a.	n.a.	No	n.a.	n.a.	n.a.	n.a.	Yes
Complications*	No	n.a.	n.a.	n.a.	n.a.	n.a.	n.a.	n.a.	n.a.	n.a.	n.a.	n.a.	n.a.	n.a.	n.a.	n.a.	n.a.	n.a.	n.a.	n.a.	n.a.	n.a.	No
Effects on Survival Rate*	No	n.a.	n.a.	n.a.	n.a.	n.a.	n.a.	n.a.	n.a.	n.a.	n.a.	n.a.	n.a.	n.a.	n.a.	n.a.	n.a.	No	n.a.	n.a.	n.a.	n.a.	n.a.
**Outcome**																							
1y survival [%]	89	80	n.a.	83	n.a.	n.a.	71	n.a.	n.a.	82	n.a.	70	n.a.	n.a.	n.a.	n.a.	n.a.	90	n.a.	90	n.a.	n.a.	n.a.
3y survival [%]	64	n.a.	44	49	47	n.a.	n.a.	n.a.	n.a.	29.5	54	36	n.a.	n.a.	68	n.a.	78	65	n.a.	45	n.a.	58	n.a.
5y survival [%]	44	33	33	27	17	n.a.	n.a.	47	40	4.5	35	n.a.	31.4	33	49	37	41	32	n.a.	45	n.a.	31	n.a.
Median survival [months]	50	24.4	34	41	39	n.a.	30	57	49	n.a.	41	n.a.	31	n.a.	40	n.a.	55	43	n.a.	11	n.a.	45.8	38
Median recurrence/disease free survival [months]	12.5	11.7	10	16	n.a.	n.a.	n.a.	12	n.a.	n.a.	n.a.	n.a.	n.a.	n.a.	12.5	39	n.a.	7.8	11	n.a.	n.a.	17.8	n.a.
Morbidity [%]	47	46.7	38	27	17	39.5	17	n.a.	34	61.4	14	45	93	n.a.	26	39	54	34.7	60	38	54.6	49	n.a.
Mortality [%]	4.5	13.3	6	5	3.6	2.3	3.4	n.a.	48	2.3	5.7	5	0	67.5	7	5	3.85	4	n.a.	n.a.	n.a.	2	n.a.

Clinical studies on interval chemotherapy during multimodal two-stage hepatectomy for patients with bilobar colorectal liver metastases

*Abbreviations*: *PVE* Portal vein embolization, *PVL* Portal vein ligation

^#^number of patients

*Yes/No

Interval chemotherapy was administered in 595 out of 1,461 patients reported in these studies, with a range of two to six treatment cycles. The fraction of patients receiving interval chemotherapy in the reported retrospective study populations was 10-100% (mean: 55%). Chemotherapy regimens used were only described in three studies (FOLFOX, FOLFIRI, FOLFIRINOX, with/ without antibodies) [12, 29, 30]. None of these studies reported on the effect of interval chemotherapy on liver hypertrophy.

### Effect of interval chemotherapy on disease control during mTSH

The effect of interventional chemotherapy on disease control during the hypertrophy phase of mTSH was reported in two studies. Muratore et al. focused on this issue in a retrospective single-center analysis of 47 mTSH cases out of 653 CRLM patients undergoing any type of liver resection (study period 1997–2009) [30]. In this cohort, 25 patients (53.2%) underwent interval chemotherapy prolonging the mean time interval between first- and second-stage resection from 1.9 to 5.2 months. Most of these patients had already received chemotherapy prior to the first liver resection (37 patients, 79%). A combination of 5-fluorouracil with either oxaliplatin or irinotecan was administered in 24 patients, the monoclonal antibodies bevacizumab and cetuximab were added to chemotherapeutic regimen in five and two patients, respectively. One patient underwent hepatic artery infusion of floxuridine. The mean dropout rate was 23% caused by intrahepatic disease progression. Overall, 25 patients (53%) showed progressive disease (PD) following first stage hepatectomy. Patients receiving and not receiving interval chemotherapy were well balanced regarding age, primary tumor stage, prior chemotherapy, and PD following first-stage resection. However, the number of CRLM at diagnosis was significantly higher in patients receiving interval chemotherapy (12.7 vs. 8.0, *p*=0.006). Interestingly, this did not result in a higher dropout rate, quite the contrary, interval chemotherapy decreased the dropout rate from 32% (7 out of 22 patients) to 16% (4 out of 25 patients), which did, however, not reach statistical significance (*p*=0.303). Interval chemotherapy resulted in partial response according to Response Evaluation Criteria in Solid Tumors (RECIST) in 20% of cases, and stable disease in 32% of cases, accounting for a disease control rate of more than 50%. Accordingly, although not statistically significant, interval chemotherapy reduced PD rate prior to second-stage resection from 59% to 48%. Unfortunately, follow-up data presented in this study were not stratified for patients receiving versus not receiving interval chemotherapy.

Giuliante et al. reported on the effect of interval chemotherapy on disease control from an Italian retrospective multicenter analysis including 130 patients (2002–2011), of which 102 patients completed second-stage resection (21.5% dropout rate) [25]. Out of these, 31 patients underwent interval chemotherapy (30.4%). Administration of interval chemotherapy was however not associated with a significantly lower risk of disease progression. As well, interval chemotherapy had no effect on 5-year survival rate (33.3 vs. 34.0%) or 5-year disease-free survival (22.8% vs. 19.2%).

### Adverse effects of interval chemotherapy during mTSH

Two studies report on adverse effects of interval chemotherapy. Muratore et al. demonstrated that interval chemotherapy neither increases the rate of complications following second-stage resection nor causes chemotherapy-induced liver damage [30].

Chavez et al. recently published data from 196 patients undergoing both stages of mTSH in five liver centers in the United States (2000–2016) [8]. In this cohort, 92% of patients received chemotherapy prior to first-stage and 60% prior to second-stage resection (118 out of 195). Surgical complications (biliary fistula/liver abscess, liver failure, thromboembolic events, wound-related events) occurred in 24% and 47% of patients post first- and second-stage resection, respectively. Administration of interval chemotherapy did not correlate with increased rates of any complications (hazard ratio 1.36). Unfortunately, details regarding chemotherapeutic regimens were not reported.

### Effects of interval chemotherapy on overall survival

Two studies describe the effect of interval chemotherapy on OS. Giuliante et al. (31 out of 102 patients who completed second stage hepatectomy) could not demonstrate an effect of interval chemotherapy on the 5-year survival (33.3% with vs. 34.0% without interval chemotherapy) or the 5-year disease-free survival (22.8% vs. 19.2%) following mTSH [25].

As well, Chavez et al. (118 out of 196 patients) did not report a positive effect of interval chemotherapy on OS (HR 1.36, 95% CI 0.85–2.17, *p*=0.202) [8].

## Discussion

Patients with bilobar CRLM generally have a poor prognosis and are frequently ineligible for curative treatment methods. However, these patients still benefit from local tumor therapy if possible [2, 4]. mTSH has been developed to provide CLRM patients that are primarily inoperable due to a high tumor burden and limited FLR with a staged, multimodal interventional treatment option, although this concept is challenged by the risk of tumor progression and by the risk of failing hypertrophy in the FLR, eventually resulting in a dropout from multimodal treatment algorithm [5]. Regimbeau et al. published the largest dataset of CRLM patients undergoing mTSH with 869 cases from the LiverMetSurvey Registry. The authors reported on a dropout rate from second-stage resection of 28% due to tumor progression. When patients could not undergo second-stage resection, the 3-/5-year OS was very limited (30%/0%), while the 3-/5-year OS of patients undergoing second-stage resection was favorable (45%/23%). The overall dropout (18%) and survival rates (53%/32% 3/5-year OS) reported in the studies identified by our literature search are slightly better, which might be due to the fact that we selected studies involving interval chemotherapy rather than reporting on the overall outcome of CRLM patients undergoing mTSH.

The so-called associating liver partition and portal vein ligation for staged hepatectomy (ALPPS) procedure was developed with the intention to improve the resection rate in CRLM patients that are initially inoperable due a limited FLR [31]. ALPPS involves the initial surgical clearance of the left lobe in combination with parenchymal transection along the intended line of resection and occlusion of the portal inflow of the right lobe, with the goal of triggering a swift growth response in the FLR. The second step consists of the resection of the deportalized right lobe and is performed after a waiting period of only one to two weeks given the strong regenerative stimulus of the initial surgical procedure. The prospectively randomized LIGRO trial compared ALPPS and TSH as treatment approaches for patients with bilobar CRLM. They demonstrated a significantly improved resection rate in the ALPPS compared to the TSH arm (92% vs. 57%), with, however, severe morbidity (43%) and mortality (6%/8%) in both arms [32]. Furthermore, the primary endpoint of the LIGRO trial was intentionally structured to favor ALPPS, at least in parts due to a stringent definition of hypertrophy failure in the TSH group, which questions the actual significance of the trial. Accordingly, mTSH is still routinely performed in several major HPB centers for the treatment of patients with bilobar CRLM with ALPPS being available as a rescue maneuver for cases with failing hypertrophy following PVE [8, 33].

A significant issue regarding mTSH is the potential for tumor progression in the FLR caused by a possible growth stimulation of preexisting micrometastases through surgical resection or hypertrophy induction. In the LIGRO trial, 16% of patients in the TSH group (8 out of 49) were unable to undergo the second-stage resection due to tumor progression, which corresponds well to the dropout range found in our review analysis. The incorporation of interval chemotherapy during mTSH has been explored as a potential strategy to enhance the completion rate of second-stage resection and thereby improving the survival outcomes of patients with bilobar CRLM. The preoperative use of chemotherapy either with or without monoclonal antibodies is well established in mCRC patients with the goal of achieving disease control or potentially reducing tumor burden [6]. Our literature search indicates that interval chemotherapy was administered in about 35% of all mTSH studies available in the current literature (21 out of 61). Although evidence is limited due to the retrospective design of the studies available in literature and heterogeneity of these studies, we conclude from our analyses that:

1. Interval chemotherapy during first and second stage of mTSH seems not to negatively affect liver growth initiated by PVE/PVL,
2. Interval chemotherapy does not increase the overall complication rate, and
3. Interval chemotherapy does not impair the oncological outcome of mTSH

Very limited evidence indicates that interval chemotherapy might improve the outcome of patients with CLRM, as shown by Muratore et al. [30]. Although not statistically significant, patients with a significantly higher tumor burden had a higher chance of undergoing second-stage resection in this series. The authors concluded that interval chemotherapy may be justified “*when the surgeon or the patient need more time before the second-stage hepatectomy*”, because interval chemotherapy neither significantly reduced nor increased the PD rate. Moreover, interval chemotherapy neither increased the rate of complications nor the chemotherapy-induced liver damage. While the latter is not supplied with data in this publication, the former has also been shown in the recent data set published by Chavez et al. [8].

However, several questions remain open. First, as stated above, a positive effect of interval chemotherapy on the outcome, i.e., PD, dropout rate from second stage resection, or overall survival of CRLM patients undergoing mTSH has not yet been proven. This might at least in parts be due to the design of the trials, which were all of retrospective nature. Moreover, OS does not well suited to judge on the benefit of interval chemotherapy because it is strongly influenced by consecutive treatment lines of the patients. The data on clinical response shown by Muratore et al. however indicate that interval chemotherapy might improve disease control during the hypertrophy phase of mTSH. A randomized clinical trial will be necessary with a primary study endpoint that is suitable to answer this question. We believe that the conduct of such a trial is justified given the current evidence, the relatively high number of patients already treated with interval chemotherapy during mTSH, and the consensus in literature that interval chemotherapy at least does not harm the patient or prevent from liver hypertrophy. Further questions to be addressed in such a trial are the actual effect of additional interval chemotherapy versus preoperative chemotherapy alone on the growth pattern of CRLM during the hypertrophy phase, i.e., are they located in the FLR or in the embolized liver, the effect on micrometastases or vanishing lesions that are not detectable by imaging technology, and the effect of molecular subtypes on the outcome of mTSH. Moreover, potential adverse effects of the addition of interval chemotherapy on liver hypertrophy or the incidence of postoperative complications are not well defined in literature and need to be addressed in such a trial.

## Conclusion

In conclusion, we hereby present the first systemic review on interval chemotherapy in patients undergoing mTSH for treatment of CRLM. Although not supported by data of randomized clinical trials, interval chemotherapy is surprisingly often used in this scenario underlining the urgent need for innovative strategies in these patients. There is no evidence in the currently available, limited literature that interval chemotherapy in addition to preoperative induction chemotherapy impairs liver hypertrophy or causes further procedure-associated complications. This calls for clinical trials to investigate the potential benefit of interval chemotherapy on disease control in CRLM patients undergoing mTSH.

## Data Availability

The data analyzed during this study is available upon reasonable request from the corresponding author.

## Abbreviations

ALPPS: Associating liver partition and portal vein ligation for staged hepatectomy
CRC: Colorectal cancer
CRLM: Colorectal liver metastases
FLR: Future liver remnant
mCRC: Metastatic colorectal cancer
mTSH: Multimodal two-stage hepatectomy
OS: Overall survival
PD: Progressive disease
PVE: Portal vein embolization
RECIST: Response Evaluation Criteria in Solid Tumors

## Data availability

The study supporting the conclusions of this article are referenced within the article.
