# Correction: Is interval chemotherapy safe and does it improve the outcome of patients with colorectal liver metastases undergoing multimodal two-stage hepatectomy? – A systematic literature review

**DOI:** 10.1186/s12885-024-13152-2

**Published:** 2024-11-11

**Authors:** Nathanael Raschzok, Simon Moosburner, Moritz Blank, Felix Krenzien, Georg Lurje, Wenzel Schöning, Igor M. Sauer, Johann Pratschke, Dominik P. Modest, Annika Kurreck

**Affiliations:** 1 Department of Surgery, Campus Charité Mitte | Campus Virchow-Klinikum, Charité – Universitätsmedizin Berlin, Corporate member of Freie Universität Berlin and Humboldt-Universität zu Berlin, Berlin, Germany; 2 Department of Hematology, Oncology, and Cancer Immunology (CCM/CVK), Charité – Universitätsmedizin Berlin, Corporate member of Freie Universität Berlin and Humboldt-Universität zu Berlin, Berlin, Germany; 3 Berlin Institute of Health at Charité - Universitätsmedizin Berlin, BIH Academy, Clinician Scientist Program, Berlin, Germany

**Correction: BMC Cancer 24, 1260 (2024)**

10.1186/s12885-024-13008-9.

Following publication of the original article [1], it was reported that the ORCID of the first author, Nathanael Raschzok, was missing. The ORCID is: https://orcid.org/0000-0001-5074-7063.

The original article [1] has been corrected.
